# Low dose human chorionic gonadotropin administration at the time of gonadotropin releasing-hormone agonist trigger versus 35 h later in women at high risk of developing ovarian hyperstimulation syndrome – a prospective randomized double-blind clinical trial

**DOI:** 10.1186/s13048-019-0483-7

**Published:** 2019-01-26

**Authors:** L. L. Engmann, B. S. Maslow, L. A. Kaye, D. W. Griffin, A. J. DiLuigi, D. W. Schmidt, D. R. Grow, J. C. Nulsen, C. A. Benadiva

**Affiliations:** 1 Division of Reproductive Endocrinology and Infertility, Department of Obstetrics and Gynecology, Center for Advanced Reproductive Services, University of Connecticut School of Medicine, Farmington, CT 06030 USA; 2 Gold Coast IVF, Woodbury, NY 11797 USA; 3 Boston IVF at the Women’s Hospital, Newburgh, IN 47630 USA; 4 Center for Advanced Reproductive Services, 2 Batterson Park Road, Farmington, CT 06032 USA

**Keywords:** Dual trigger, Leuprolide trigger, Gonadotropin-releasing hormone agonist, Ovarian hyperstimulation syndrome, OHSS, Low dose human chorionic gonadotropin, hCG and agonist trigger, Luteal support

## Abstract

**Background:**

Ovarian hyperstimulation syndrome remains a serious complication during in vitro fertilization cycles if high dose human chorionic gonadotropin (hCG) is used to trigger ovulation in high responder patients. Though much of this risk is mitigated with trigger using gonadotropin releasing-hormone (GnRH) agonist alone, it may result in lower birth rates. GnRH-agonist trigger and adjuvant low dose hCG has been proposed to improve birth rates, but timing of this hCG support to corpus luteum function has never been fully described. In this randomized, prospective trial, we explore differences in live birth rates and incidence of ovarian hyperstimulation syndrome (OHSS) in high-responder patients undergoing in vitro fertilization (IVF) receiving low dose hCG at the time of GnRH-agonist (dual trigger) or hCG adjuvant at the time of oocyte retrieval. Does the timing of hCG support make a difference?

**Results:**

Thirty-four subjects high-responder patients were randomized to receive low-dose hCG at the time of GnRH-agonist trigger (Group 1) and 37 received low-dose hCG at the time of oocyte retrieval (Group 2). There were no differences in the baseline characteristics and outcome of ovarian stimulation between the two groups. There were no differences in the live birth rates between Group 1 and Group 2 by intention-to-treat (14/34, 41.2% versus 21/37, 56.8%, *p* = 0.19) or per-protocol (14/26, 53.8% versus 19/31, 61.3%, *p* = 0.57) analyses. There was a slightly higher incidence of OHSS in Group 2 compared to Group 1 although the difference was not statistically significant (3/31, 9.7% versus 1/26, 3.8%). All the cases of OHSS in Group 2 were moderate while the one case of OHSS in Group 1 was mild.

**Conclusions:**

For high responder patients receiving GnRH-agonist trigger, low dose hCG supplementation allowed high pregnancy rates after fresh embryo transfer, regardless of whether it was given at the time of trigger or at oocyte retrieval. Dual trigger may be preferable to reduce the risk of OHSS.

## Background

Ovarian hyperstimulation syndrome (OHSS) is a complication of ovarian stimulation during in vitro fertilization (IVF) cycles. Although the severe form is uncommon, it may result in significant morbidity including hospitalization. Induction of oocyte maturation using gonadotropin releasing hormone (GnRH) agonist instead of human chorionic gonadotropin (hCG) has been shown to be effective in preventing the development of OHSS [1–3]. The short duration of the luteinizing hormone (LH) surge induced by GnRH agonist administration results in defective corpus luteum (CL) formation [4, 5]. This culminates in abnormal secretion of growth factors resulting in prevention of OHSS [6, 7].

However, the unwanted consequence of the defective corpora lutea function is lower pregnancy rates after GnRH agonist trigger [8, 9]. Elective cryopreservation of all oocytes or embryos after GnRH agonist trigger and transfer in a subsequent cycle maintains excellent pregnancy rates [10–12]. Since fresh embryo transfers are still preferable for some patients, it is paramount to design protocols that are safe and effective in optimizing pregnancy rates after GnRH agonist trigger whilst reducing the risk of OHSS development.

Studies have shown that the use of intensive luteal phase support with estradiol (E_2_) and intramuscular (IM) progesterone (P) improves pregnancy rates, but only in a subgroup of patients [1, 13, 14]. Adjuvant low dose hCG along with GnRH agonist trigger protocols have been suggested as a modality to further improve pregnancy rates. Adjuvant low dose hCG can be given at the time of trigger (‘dual trigger’) as either a variable dose ranging from 1000 to 2500 IU based on OHSS risk and body weight [15, 16] or a fixed dose of 1000 IU [13]. It is thought that administration of low dose hCG rescues a small number of corpora lutea and may result in increased pregnancy rates over GnRH agonist trigger alone [13]. In a retrospective review of our GnRH agonist trigger experience in high risk patients with peak E_2_ < 4000 pg/mL, we found a higher live birth rate after dual GnRH agonist and hCG 1000 IU trigger compared with GnRH agonist trigger alone (52.9% versus 30.9%) [13]. Alternatively, low dose hCG can be given at the time of oocyte retrieval and a dose of 1500 IU has been shown to improve pregnancy rates in high responders after GnRH agonist trigger [17–19].

Given the physiologic plausibility of the dual trigger (GnRH agonist + 1000 IU hCG) rescuing a small number of corpora lutea, we hypothesize that it would improve pregnancy rates, while decreasing the risk of OHSS, compared to administration of adjuvant low dose hCG of 1500 IU at the time of oocyte retrieval. The minimal dose of hCG necessary to support the luteal phase after GnRH agonist trigger has not been established, however, hCG of 1000 IU is so far the lowest dose reported to be safe and effective [13]. The use of adjuvant hCG of any dose after GnRH agonist trigger may result in an increased risk of OHSS and a high proportion of severe OHSS have been reported after the use of hCG 1500 at the time of oocyte retrieval [20]. Although several cases of severe OHSS were reported after the use of dual trigger in high responders, almost all the cases occurred in women with peak E_2_ levels of > 4000 pg/ml [21]. Moreover, there has never been a comparison done between the two protocols in high risk patients.

We designed a prospective, randomized, double-blind, placebo controlled trial to explore any differences between two well established protocols, dual trigger with low dose hCG plus GnRH agonist versus low dose hCG administration 35 h after GnRH agonist trigger at the time of oocyte retrieval in women at risk of OHSS with peak serum E_2_ o < 4000 pg/mL.

## Methods

### Study design

We have previously reported the study design, rationale and protocol overview [22]. This study was a single university-affiliated center prospective randomized double-blind placebo controlled trial. The subjects were recruited from March 2013 to December 2015. Approval for this study was obtained from the university Institutional Review Board. Low dose hCG was used under an Investigational New Drug (IND) application to the Food and Drug Administration (IND# 113472). The trial was registered at clinicaltrials.gov (NCT#: NCT01815138). The study had to be terminated prematurely because of difficulties with recruitment associated with changes in staff and study site location.

### Participants

Subjects who were considered at high risk for the development of OHSS by the following criteria were recruited for the study: (1) age ≥ 18 years and < 40 years, (2) normal serum day 3 follicle stimulating hormone (FSH) < 10 mIU/mL (3) diagnosis of polycystic ovarian syndrome (PCOS) or ≥ 12 antral follicles in at least one ovary or serum AMH > 3.5 ng/ml; or (4) patients with a history of high response to gonadotropins. PCOS was defined according to the Rotterdam consensus guidelines [23]. Previous high response was defined as history of cycle cancellation due to high response or history of significant OHSS after controlled ovarian stimulation (COS).

To meet final inclusion criteria for the study, all subjects recruited had to have > 14 follicles of over 11 mm in diameter [24] and peak E_2_ levels < 4000 pg/mL on the day of trigger of oocyte maturation. Subjects with hypothalamic dysfunction would not be expected to respond to GnRH agonist trigger, and were excluded from the study. On the day of trigger, subjects who had < 14 follicles at > 11 mm in diameter were thought to be at low risk for OHSS to benefit from a GnRH agonist trigger and were excluded from the study. Subjects with peak E_2_ levels ≥4000 pg/mL were considered too high risk of OHSS for adjuvant hCG and were also excluded from the study.

### Randomization

Subjects who were recruited and consented for the study were randomly assigned to one of the two groups in a ratio of 1:1 by means of computer-generated random numbers. Randomization of subjects into the appropriate study groups was performed by the University Institutional Drug Services (IDS) with a series of consecutively numbered sealed opaque envelopes, therefore concealing the sequence of allocation. The study was blinded to both the clinical staff and the subjects.

Randomization occurred when subject’s leading follicle was 14 mm in mean diameter. This strategy allowed enough time for the IDS to randomize the patient and prepare the study medications for prompt delivery at the time of trigger.

The subjects were randomly assigned to two groups: Group 1 subjects received GnRH agonist (leuprolide acetate 1 mg) and low dose hCG 1000 IU at the time of trigger and then received the placebo at oocyte retrieval (35 h after trigger). Group 2 subjects received GnRH agonist (leuprolide acetate 1 mg) and placebo at the time of trigger and then received low dose hCG 1500 IU at the time of oocyte retrieval (35 h after trigger).

### Study protocol

Eligible subjects were recruited by the research staff and consented for the study prior to commencing an IVF cycle. The treatment protocol has previously been described [22]. In brief, COS was achieved using a step-down protocol of recombinant FSH (Follistim; Organon USA Inc., Roseland, NJ, United States) with or without human menopausal gonadotropin (hMG; Menopur; Ferring Pharmaceuticals, Parsippany, NJ, United States) in a total dose of 112–225 IU/day. The starting gonadotropin dose was based on age, body mass index (BMI), day 3 FSH, AMH, antral follicle count (AFC) and prior response to gonadotropins, and was determined by their treating physician prior to enrollment in the study. Subjects’ response was monitored during the IVF cycle using serial transvaginal ultrasounds for follicular measurements and serum E_2_, P and LH levels. The dose of gonadotropin was adjusted according to the patient’s response. GnRH antagonist (Ganirelix; Organon USA Inc., Roseland, NJ, United States) was started at 0.25 mg subcutaneously daily once the leading follicle reached ≥14 mm in diameter or serum E_2_ > 350 pg/mL and continued until the day of oocyte maturation trigger. Subjects were triggered with leuprolide acetate 1 mg (Lupron; TAP Pharmaceuticals, North Chicago, IL, United States) subcutaneously with hCG 1000 IU or placebo subcutaneously when at least 3 follicles reached ≥17 mm in mean diameter. Serum LH, E_2,_ P levels were assessed the day after trigger to ensure adequate LH surge response to GnRH agonist trigger [25]. Transvaginal ultrasound guided oocyte retrieval was performed 35 h after the trigger injection and hCG 1500 IU or placebo was administered. Embryo transfer was performed 3 (cleavage stage) or 5 (blastocyst stage) days after oocyte retrieval based on embryo quality.

Subjects started IM P 50 mg daily and transdermal E_2_ patches 0.3 mg every other day (Vivelle-Dot; Novartis Pharmaceuticals, East Hanover, NJ, United States) from the day after oocyte retrieval until a negative pregnancy test or 10 weeks gestation. Serum P and E_2_ levels were measured on day five (+ 5) and nine (+ 9) after GnRH agonist trigger and at the time of pregnancy test, and then weekly until 10 weeks gestation. The dose of E_2_ patches was increased, if necessary, up to a maximum of 0.4 mg every other day and/or addition of oral micronized E_2_ (Estrace; Bristol-Myers, Princeton, NJ, United States) 2 mg by mouth twice daily to maintain serum E_2_ levels > 200 pg/mL. If needed, IM P was increased to a maximum dose of 75 mg IM daily and/or vaginal progesterone gel 90 mg daily (Crinone 8%; Juniper Pharmaceuticals) was added to maintain serum P levels > 20 ng/mL.

All subjects were evaluated 9 days after GnRH agonist trigger (mid-luteal) for signs and symptoms of OHSS as well as ultrasound measurement of the ovarian volumes and presence of fluid in pelvis or abdomen. The diagnosis of OHSS was based on the criteria by Golan et al. [26]. Ovarian volume was calculated using the prolate ellipsoid formula (V = D1 x D2 x D3 × 0.523). The mean ovarian volume was defined as the average volume of the two ovaries ((V1+ V2)/2).

### Sample size

Sample size was originally calculated based on previously reported live birth rate for each of the two selected protocols. A previous study reported a 24% delivery rate following hCG 1500 IU administration 35 h after GnRH agonist trigger [18]. However, while this was not significantly different from standard hCG trigger live birth rate of 31% [18], it was much higher than their previous reported clinical pregnancy rate of 6% after GnRH agonist trigger alone [8]. In a retrospective review of our GnRH agonist trigger experience in high risk patients with peak E_2_ < 4000 pg/mL, we reported a higher live birth rate after dual trigger (GnRH agonist + hCG 1000 IU) compared with GnRH agonist trigger alone (52.9% versus 30.9%) [13].

Based on a 0.05 two-sided significance level, we calculated that a sample size of 41 subjects in each group will provide 80% power to detect a significant difference in the live birth rates between the Group 2 proportion of 0.240 and a Group 1 proportion of 0.529.

### Outcomes

The original primary outcome was live birth rate. Secondary outcomes included clinical pregnancy and ongoing pregnancy rates and OHSS. Other study endpoints included implantation and miscarriage rates and mid-luteal ovarian volumes and luteal phase hormone profile. Clinical pregnancy rate was defined as the presence of a gestational sac with a fetal pole and fetal cardiac activity on transvaginal ultrasound. Ongoing pregnancy rate was defined as the presence of a gestational sac with a fetal pole and fetal cardiac activity on transvaginal ultrasound which proceeds beyond 12 weeks gestation. In view of the fact that the study was underpowered, the we just report the findings of the study.

### Statistical analyses

All randomized subjects were included in the analysis of the primary efficacy endpoint (intention-to-treat analysis). For analyses of other outcome variables, only subjects who were randomized and received the study medication and underwent a fresh embryo transfer were included (per-protocol analysis).

Statistical analyses were performed using the Statistical Package for the Social Sciences (Release 24.0; SPSS, Chicago, IL). Chi-square or Fisher exact tests were used for categorical variables where appropriate. Independent sample t-test or Mann-Whitney U was used for continuous variables based on whether the data was normally distributed. A binomial logistic regression was performed evaluating live birth rate between Group 1 and Group 2 controlling for covariates including age, BMI, number of oocytes retrieved, number of embryos transferred and day of embryo transfer (day 3 or day 5).

Data are presented as mean ± SD unless otherwise stated. All *P* values quoted are two-sided, and values < 0.05 were taken to indicate statistical significance.

## Results

### Participant flow

**Fig. 1:**
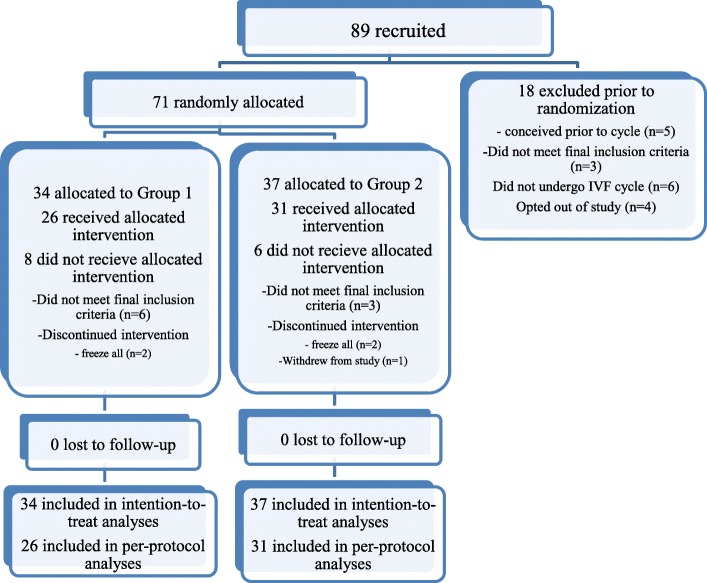
Flowchart of participants in the study

The study flow is presented in Fig. 1. A total of 89 subjects were recruited and consented. Eighteen subjects were excluded prior to randomization. Seventy-one subjects were randomized (Group 1 = 34, Group 2 = 37). Fourteen subjects commenced treatment but discontinued intervention after randomization. They were included in the intention-to-treat analysis but excluded in the per-protocol analysis (Group 1 = 8, Group 2 = 6). A total of 57 subjects completed study protocol and were included in the per-protocol analysis (Group 1 = 26, Group 2 = 31).

### Baseline and cycle characteristics

The two groups were comparable in mean age, BMI, day 3 FSH, AFC, proportion of patients with PCOS and primary cause of infertility (Table 1). The mean AMH in the two groups was 8.3 ± 5.8 ng/ml in Group 1 and 8.4 ± 4.9 ng/ml in Group 2. The majority of patients in both groups had serum AMH > 3.5 ng/ml, confirming that both groups were comprised of subjects with high ovarian reserve and therefore potential high responders.

**Table 1:** Baseline characteristics

	Group 1 (*n* = 26)	Group 2 (*n* = 31)	*P*-value
Age	31.3 ± 3.3	32.2 ± 3.4	0.31
Parity (n)	0.1 ± 0.4	0.1 ± 0.4	0.94
BMI (kg/m^2^)	26.3 ± 4.8	27.4 ± 5.5	0.40
Antral follicle count (n)	22.2 ± 7.8	24.3 ± 12.4	0.48
Antral follicle count > 12 (n, %)	24/26 (92.3)	24/31 (77.4)	0.12
AMH (ng/ml)	8.3 ± 5.8	8.4 ± 4.9	0.98
AMH > 3.5 (n, %)	23/26 (88.5)	30/31 (96.8)	0.22
Baseline serum FSH (IU/L)	5.6 ± 1.7	5.2 ± 1.6	0.39
PCOS (n, %)	8/26 (30.8)	8/31 (25.8)	0.67
Primary cause of infertility (n, %)			0.22
Anovulation	20/26 (76.9)	18/31 (58.1)	
Male Factor	3/26 (11.5)	2/31 (6.5)	
Tubal disease	1/26 (3.8)	2/31 (6.5)	
Unexplained	1/26 (3.8)	8/31 (25.8)	
Others	1/26 (3.8)	1/31 (3.2)	

The outcome of ovarian stimulation is presented in Table 2. The duration of ovarian stimulation and total dose of gonadotropin required were comparable in both groups. A mean of 18.4 oocytes were retrieved in each group, validating that both groups were high responders. The proportion of mature oocytes, fertilization rates, proportion of patients who had blastocysts transferred, number of embryos frozen and proportion of embryos frozen were similar both two groups. The number of embryos transferred was not different between Group 1 and Group 2 (1.5 ± 0.5 versus 1.7 ± 0.5, *p* = 0.10).

**Table 2:** Outcome of ovarian stimulation

	Group 1 (*n* = 26)	Group 2 (*n* = 31)	*P*-value
Total dose of gonadotropins (IU)	1553 ± 431	1546 ± 527	0.96
Duration of ovarian stimulation (days)	8.6 ± 1.7	8.6 ± 1.4	0.83
Oocytes (n)	18.4 ± 5.9	18.4 ± 7.7	0.99
Proportion of mature oocytes (%)	85.2 ± 10.2	79.5 ± 16.9	0.18
Fertilization rate (%)	83.3 ± 11.4	80.3 ± 12.6	0.38
Embryos transferred (n)	1.5 ± 0.5	1.7 ± 0.5	0.10
Blastocyst transfer (n, %)	24/26 (92.3)	26/31 (83.9)	0.44
Embryos frozen (n)	3.3 ± 3.0	3.4 ± 3.4	0.91
Patients with frozen embryos (n, %)	21/26 (80.8)	23/31 (74.2)	0.56

### Hormone profile

The luteal phase hormone profiles are shown in Table 3. There were no differences between the two groups in the serum E_2_ or P on the day of trigger. There was a rise in serum LH approximately 12 h after GnRH agonist trigger in both Group 1 (from 2.1 ± 1.5 to 75.0 ± 47.3 mIU/mL) and Group 2 (from 2.3 ± 2.3 to 82.3 ± 50.0 mIU/mL), and the differences between the two groups were not statistically significant. All patients in the study had optimal responses to the GnRH agonist trigger, defined as serum P of > 3.5 ng/mL and LH > 15 IU/L post-trigger [25] and no retrieval was cancelled for lack of surge.

**Table 3:** Hormonal profile

	Group 1 (*n* = 26)	Group 2 (*n* = 31)	*P*-value
Serum E_2_ day of trigger (pg/mL)	2479.7 ± 1345.0	2373.0 ± 892.2	0.77
Serum P day of trigger (ng/mL)	1.0 ± 0.4	0.9 ± 0.3	0.54
Serum LH day of trigger (mIU/mL)	2.1 ± 1.5	2.3 ± 2.3	0.76
Serum LH post trigger (mIU/mL)	75.0 ± 47.3	82.3 ± 50.0	0.57
Serum P post trigger (ng/mL)	5.5 ± 3.6	4.8 ± 2.3	0.61
Serum E_2_ 5 days after trigger (pg/mL)	1576.0 ± 800.3	1914.0 ± 914.8	0.14
Serum E_2_ 9 days after trigger (pg/mL)	468.8 ± 304.0	895.6 ± 456.8	< 0.01
Serum P 5 days after trigger (ng/mL)	122.1 ± 222.2	103.1 ± 42.7	0.67
Serum P 9 days after trigger (ng/mL)	29.9 ± 29.7	30.1 ± 19.1	0.98

There were no significant differences between the two groups in the serum E_2_ or P levels five days after trigger. The mean serum E_2_ level 9 days after trigger was significantly higher in Group 2 compared to Group 1, although the serum P levels were not different.

### Outcome measures

There were no differences in the live birth rates between Group 1 and Group 2 by intention-to-treat (14/34, 41.2% versus 21/37, 56.8%, *p* = 0.19) or per-protocol (14/26, 53.8% versus 19/31, 61.3%, *p* = 0.57) analyses (Table 4). Subjects who were randomized but did not have a transfer including those who froze all embryos were considered not pregnant in the intention to treat analysis. Upon adjusting for potential confounders using logistic regression analysis, we obtained an adjusted odds ratio for live birth of 0.65 ([95% CI 0.21–2.01], *p* = 0.45) when comparing Group 1 to Group 2, confirming that there was no difference between the groups. There were no differences in clinical or ongoing pregnancy rates or miscarriage rates between the two groups. The twin clinical pregnancy rate was 13.3% (2/15) and 31.8% (7/22) in Groups 1 and 2 respectively. There was one case of ectopic pregnancy in each group.

**Table 4:** Outcome measures

	Group 1	Group 2	*P*-value
Primary end points			
Live Birth Rate (intention to treat), n (%)	14/34 (41.2)	21/37 (56.8)	0.19
Live Birth Rate (per protocol), n (%)	14/26 (53.8)	19/31 (61.3)	0.57
Secondary endpoint (per protocol)			
Clinical Pregnancy rate, n (%)	15/26 (57.7)	22/31 (71.0)	0.29
Ongoing Pregnancy Rate, n (%)	15/26 (57.7)	19/31 (61.3)	0.79
Mild/Moderate OHSS, n (%)	1/26 (3.8)	3/31 (9.7)	0.62
Other end points (per protocol)			
Mid-luteal ovarian volume (cm^3^)	77.6 ± 57.6	100.1 ± 60.4	0.13
Implantation rate, n (%)	17/38 (44.7)	30/58 (57.6)	0.44
Positive pregnancy rate, n (%)	19/26 (73.1)	25/31 (80.6)	0.49
Overall Miscarriage rates, n (%)	5/19 (26.3)	6/25 (24)	0.86
Clinical Miscarriage rate, n (%)	1/15 (6.7)	3/22 (13.6)	0.63

The incidence of OHSS was 3.8% (1/26) in Group 1 which was not significantly different from the incidence in Group 2 of 9.7% (3/31). There was one case of mild OHSS and no cases of moderate or severe OHSS in Group 1 and three cases of moderate OHSS in Group 2. Additionally, the mean ovarian volume was smaller in Group 1 than Group 2 although the difference was not statistically significant (77.6 ± 57.6 cm^3^ versus 100.1 ± 60.4 cm^3^, *p* = 0.13).

## Discussion

The findings of this study suggest there are no differences in live birth rates between subjects who received low dose hCG 1000 IU at the time of GnRH agonist trigger and hCG 1500 given at the time of oocyte retrieval after GnRH agonist trigger. However, this study is underpowered in detecting differences in live birth rates and therefore the results should be interpreted with caution. More cases of moderate OHSS occurred in women who received low dose hCG at time of oocyte retrieval, however, the differences were not significant.

In view of the reported low pregnancy rates after fresh embryo transfer [8, 9] and excellent pregnancy rates following frozen thawed embryo transfer cycles after either hCG or GnRH agonist trigger [11, 27, 28], elective cryopreservation of all oocytes or embryos has become an attractive option after GnRH agonist trigger. However, there are some patients who may still prefer fresh embryo transfers because of financial pressures, insurance coverage, or personal considerations. In addition, not all IVF clinics have established successful embryo cryopreservation programs that can provide the same results reported by the more experienced centers. It is therefore incumbent upon us to optimize our luteal phase protocols to improve implantation rates and enable fresh embryo transfers while still taking advantage of GnRH agonist trigger to decrease the risk of OHSS.

Corpus luteum dysfunction and early CL demise is the underlying reason for the significantly low pregnancy rates after GnRH agonist trigger. Intensive steroid luteal support using exogenous E_2_ and P supplementation improves pregnancy rates after GnRH agonist trigger [1, 16, 29, 30]. However, the benefit from intensive luteal support does not extend to all patients triggered with GnRH agonist. Women with low serum LH and serum E_2_ < 4000 pg/mL have a lower pregnancy rate compared with women with peak E_2_ over 4000 pg/mL, despite intensive steroid hormone support [14].

Adjuvant low-dose hCG has been proposed as an option to rescue a few CL to improve luteal phase function and improve pregnancy rates without increasing the risk of OHSS [13, 15–18, 31, 32]. Our selection of the time and dose of adjuvant hCG administration after GnRH agonist trigger was based on two common approaches previously described to improve pregnancy rates [32]. The purpose of this study was to determine if one protocol has superior pregnancy rates without increasing the risk of OHSS.

We selected a fixed dose of adjuvant hCG 1000 IU at the time of GnRH agonist trigger for the study group (Group 1) based on the assumption that it may be high enough to rescue a few of the corpora lutea to optimize conception rates, but not too high to rescue excessive numbers of CL, thereby reducing the risk of significant OHSS development [13]. The use of adjuvant hCG 1500 IU administered 35 h after GnRH agonist trigger is well supported in the literature with similar pregnancy rates compared with standard dose hCG trigger [18, 31, 32], and was therefore selected as the control group (Group 2). Physiologically, this approach takes advantage of the fact that luteinized granulosa cells obtained at the time of oocyte retrieval after GnRH agonist trigger are viable and have the same potential for hCG rescue as cells obtained after hCG trigger [33]. Therefore, despite GnRH agonist trigger, the corpus luteum responds to a bolus of hCG administration 35 h later. A previous study had compared GnRH agonist trigger and adjuvant low dose hCG 1500 IU 12 h after trigger versus 35 h later and showed a higher clinical pregnancy rate when given 35 h after trigger [17].

Our study demonstrates that both the dose and timing of the two adjuvant hCG protocols were optimal, as evidenced by the fact that there were no significant differences in pregnancy rates between the two protocols. The addition of any dose of hCG may result in a higher incidence of OHSS [20] and therefore it is essential to select patients who will benefit from adjuvant low dose hCG without increasing significantly their risk of developing OHSS [34]. Those at highest risk of OHSS including patients with > 20 follicles or peak serum E_2_ > 4000 pg/ml, may be candidates for GnRH agonist trigger alone and freeze all oocytes or embryos, since such patients may have a higher incidence of significant OHSS after low dose adjuvant hCG use [21, 34]. While there were no statistically significant differences in the incidence of OHSS between the two groups, all the subjects with OHSS who received hCG at the time of oocyte retrieval (Group 2) had moderate OHSS in contrast to only one case of mild OHSS in the dual trigger group (Group 1). Moreover, the mid-luteal ovarian volume was smaller in the dual trigger group, although the differences were not significant. Therefore, for the prevention of OHSS, dual trigger with adjuvant hCG 1000 IU at the time GnRH agonist trigger may be preferable without compromising success. Further studies can explore whether this may be due to the lower dose of hCG or the earlier timing of administration.

The routine use of dual trigger with low dose hCG may be advantageous since it may ensure retrieval of appropriate number of mature oocytes in cases of failed endogenous response to GnRH agonist trigger and therefore this approach has been used even in oocyte donors and patients who may be freezing all their embryos [35]. In this study, there was no advantage of the dual trigger approach in oocyte yield since all patients had an optimal endogenous hormone rise to the GnRH agonist trigger. Hence, the number of oocytes as well as proportion of oocytes obtained were comparable in the two protocols.

A major strength of this study is the prospective randomized nature of the trial. Moreover, the study was double blinded with the use of placebo and therefore the subjects, physicians and researchers were blinded to the assigned treatment groups. All the patients in the study returned 9 days after trigger for an assessment of OHSS, allowing an objective assessment of ovarian volume as well as symptoms of OHSS. The main limitations of the study include the relatively small sample size and also the high number of patients excluded after randomization. We did a post-hoc power calculation which showed that a sample size of 85 subjects will be required to detect a significant difference in the live birth rates per intention to treat between Group 1 proportion of 0.41 and a Group 2 proportion of 0.56. The study is therefore underpowered to determine differences between the two groups. In order to allow enough time for low dose hCG and placebo preparation and delivery during ovarian stimulation but before the day of trigger, we randomized subjects prior to meeting all the final inclusion criteria. It is also possible that the different days and dosages of adjuvant hCG administration do not truly allow direct comparison of dose and timing of adjuvant hCG use. However, we selected these two regimens to perform a comparison between two well established protocols.

Although this study may be underpowered to determine differences between the two groups, it has been argued that some information is better than none and that a small amount of inconclusive information may contribute to a larger systemic review [36, 37], particularly when there are no previous trials addressing this specific topic. When sample size is constrained by logistic or recruitment barriers, it has been suggested that reporting the results within these constraints is a sensible choice [36, 37].

Despite the limitations, our study demonstrates that adjuvant low dose hCG administered either at the time of trigger or 35 h later results in excellent pregnancy rates. This demonstrates that either protocol may be a reliable option for patients at risk of OHSS who desire a fresh transfer. While there were no significant differences between the two protocols, a lower dose of hCG given earlier in the dual trigger protocol may be preferable in order to reduce the risk of OHSS development.

## Abbreviations

AFC: Antral Follicle Count
AMH: Anti-mullerian hormone
BMI: Body Mass Index
CL: Corpus luteum
Dual Trigger: Low dose hCG at the time of GnRH-agonist
E_2_: Estradiol
FSH: Follicle stimulating hormone
GnRH: Gonadotropin releasing-hormone
hCG: Human chorionic gonadotropin
hMG: Human menopausal gonadotropin
IDS: Institutional Drug Services
IM: Intramuscular
IND: Investigational New Drug
IV: In vitro fertilization
OHSS: Ovarian hyperstimulation syndrome
P: Progesterone
PCOS: Polycystic ovarian syndrome

## References

[1] Engmann L, DiLuigi A, Schmidt D, Nulsen J, Maier D, Benadiva C (2008). The use of gonadotropin-releasing hormone (GnRH) agonist to induce oocyte maturation after cotreatment with GnRH antagonist in high-risk patients undergoing in vitro fertilization prevents the risk of ovarian hyperstimulation syndrome: a prospective rando. Fertil Steril.

[2] Humaidan P, Kol S, Papanikolaou EG (2011). GnRH agonist for triggering of final oocyte maturation: time for a change of practice?. Hum Reprod Update.

[3] Youssef MA, Van der Veen F, Al-Inany HG, Mochtar MH, Griesinger G, Nagi Mohesen M, et al. Gonadotropin-releasing hormone agonist versus HCG for oocyte triggering in antagonist-assisted reproductive technology. Cochrane Database Syst Rev. 2014;10:CD008046. In: Youssef MA, editor. 2014/11/02. Chichester, UK: John Wiley & Sons, Ltd. 10.1002/14651858.CD008046.pub4 PMC10767297 25358904

[4] Hoff JD, Quigley ME, Yen SS (1983). Hormonal dynamics at midcycle: a reevaluation. J Clin Endocrinol Metab.

[5] Chandrasekher YA, Brenner RM, Molskness TA, Yu Q, Stouffer RL (1991). Titrating luteinizing hormone surge requirements for ovulatory changes in primate follicles. II. Progesterone receptor expression in luteinizing granulosa cells. J Clin Endocrinol Metab.

[6] Miller I, Chuderland D, Ron-El R, Shalgi R, Ben-Ami I (2015). GnRH agonist triggering modulates PEDF to VEGF ratio inversely to hCG in granulosa cells. J Clin Endocrinol Metab.

[7] Cerrillo M, Rodríguez S, Mayoral M, Pacheco A, Martínez-Salazar J, Garcia-Velasco JA (2009). Differential regulation of VEGF after final oocyte maturation with GnRH agonist versus hCG: a rationale for OHSS reduction. Fertil Steril.

[8] Humaidan P, Ejdrup Bredkjær H, Bungum L, Bungum M, Grøndahl ML, Westergaard L (2005). GnRH agonist (buserelin) or hCG for ovulation induction in GnRH antagonist IVF/ICSI cycles: a prospective randomized study. Hum Reprod.

[9] Kolibianakis EM, Schultze-Mosgau A, Schroer A, van Steirteghem A, Devroey P, Diedrich K (2005). A lower ongoing pregnancy rate can be expected when GnRH agonist is used for triggering final oocyte maturation instead of HCG in patients undergoing IVF with GnRH antagonists. Hum Reprod.

[10] Griesinger G, Kolibianakis EM, Papanikolaou EG, Diedrich K, Van Steirteghem A, Devroey P (2007). Triggering of final oocyte maturation with gonadotropin-releasing hormone agonist or human chorionic gonadotropin. Live birth after frozen-thawed embryo replacement cycles. Fertil Steril.

[11] Griesinger G, Berndt H, Schultz L, Depenbusch M, Schultze-Mosgau A (2010). Cumulative live birth rates after GnRH-agonist triggering of final oocyte maturation in patients at risk of OHSS: a prospective, clinical cohort study. Eur J Obstet Gynecol Reprod Biol.

[12] Herrero L, Pareja S, Losada C, Cobo AC, Pellicer A, Garcia-Velasco JA (2011). Avoiding the use of human chorionic gonadotropin combined with oocyte vitrification and GnRH agonist triggering versus coasting: a new strategy to avoid ovarian hyperstimulation syndrome. Fertil Steril.

[13] Griffin D, Benadiva C, Kummer N, Budinetz T, Nulsen J, Engmann L (2012). Dual trigger of oocyte maturation with gonadotropin-releasing hormone agonist and low-dose human chorionic gonadotropin to optimize live birth rates in high responders. Fertil Steril.

[14] Kummer N, Benadiva C, Feinn R, Mann J, Nulsen J, Engmann L (2011). Factors that predict the probability of a successful clinical outcome after induction of oocyte maturation with a gonadotropin-releasing hormone agonist. Fertil Steril.

[15] Shapiro BS, Daneshmand ST, Garner FC, Aguirre M, Thomas S (2008). Gonadotropin-releasing hormone agonist combined with a reduced dose of human chorionic gonadotropin for final oocyte maturation in fresh autologous cycles of in vitro fertilization. Fertil Steril.

[16] Shapiro BS, Daneshmand ST, Garner FC, Aguirre M, Hudson C (2011). Comparison of “triggers” using leuprolide acetate alone or in combination with low-dose human chorionic gonadotropin. Fertil Steril.

[17] Humaidan P, Bungum L, Bungum M, Andersen CY (2006). Rescue of corpus luteum function with peri-ovulatory HCG supplementation in IVF/ICSI GnRH antagonist cycles in which ovulation was triggered with a GnRH agonist: a pilot study. Reprod BioMed Online.

[18] Humaidan P, Ejdrup Bredkjær H, Westergaard LG, Yding Andersen C (2010). 1,500 IU human chorionic gonadotropin administered at oocyte retrieval rescues the luteal phase when gonadotropin-releasing hormone agonist is used for ovulation induction: a prospective, randomized, controlled study. Fertil Steril.

[19] Humaidan P, Polyzos NP, Alsbjerg B, Erb K, Mikkelsen AL, Elbaek HO (2013). GnRHa trigger and individualized luteal phase hCG support according to ovarian response to stimulation: two prospective randomized controlled multi-Centre studies in IVF patients. Hum Reprod.

[20] Seyhan A, Ata B, Polat M, Son W-Y, Yarali H, Dahan MH (2013). Severe early ovarian hyperstimulation syndrome following GnRH agonist trigger with the addition of 1500 IU hCG. Hum Reprod.

[21] O’Neill KE, Senapati S, Maina I, Gracia C, Dokras A (2016). GnRH agonist with low-dose hCG (dual trigger) is associated with higher risk of severe ovarian hyperstimulation syndrome compared to GnRH agonist alone. J Assist Reprod Genet.

[22] Griffin D, Benadiva C, Budinetz T, Sueldo C, DiLuigi A, Nulsen J (2017). The dual trigger study: rationale and study design of a prospective double-blind randomized clinical trial comparing pregnancy rates after co-administration of low dose hCG at the time of GnRH agonist trigger or 35 h later for the prevention of OHSS. Contemp Clin Trials Commun.

[23] The Rotterdam ESHRE/ASRM-sponsored PCOS consensus workshop group (2004). Revised 2003 consensus on diagnostic criteria and long-term health risks related to polycystic ovary syndrome (PCOS). Hum Reprod.

[24] Papanikolaou EG, Pozzobon C, Kolibianakis EM, Camus M, Tournaye H, Fatemi HM (2006). Incidence and prediction of ovarian hyperstimulation syndrome in women undergoing gonadotropin-releasing hormone antagonist in vitro fertilization cycles. Fertil Steril.

[25] Kummer NE, Feinn RS, Griffin DW, Nulsen JC, Benadiva CA, Engmann LL (2013). Predicting successful induction of oocyte maturation after gonadotropin-releasing hormone agonist (GnRHa) trigger. Hum Reprod.

[26] Golan A, Ron-el R, Herman A, Soffer Y, Weinraub Z, Caspi E (1989). Ovarian hyperstimulation syndrome: an update review. Obstet Gynecol Surv.

[27] Shapiro BS, Daneshmand ST, Garner FC, Aguirre M, Hudson C, Thomas S (2011). Evidence of impaired endometrial receptivity after ovarian stimulation for in vitro fertilization: a prospective randomized trial comparing fresh and frozen-thawed embryo transfers in high responders. Fertil Steril.

[28] Shapiro BS, Daneshmand ST, Garner FC, Aguirre M, Hudson C, Thomas S (2011). Evidence of impaired endometrial receptivity after ovarian stimulation for in vitro fertilization: a prospective randomized trial comparing fresh and frozen–thawed embryo transfer in normal responders. Fertil Steril.

[29] Iliodromiti S, Lan VT, Tuong HM, Tuan PH, Humaidan P, Nelson SM (2013). Impact of GnRH agonist triggering and intensive luteal steroid support on live-birth rates and ovarian hyperstimulation syndrome: a retrospective cohort study. J Ovarian Res.

[30] Imbar T, Kol S, Lossos F, Bdolah Y, Hurwitz A, Haimov-Kochman R (2012). Reproductive outcome of fresh or frozen-thawed embryo transfer is similar in high-risk patients for ovarian hyperstimulation syndrome using GnRH agonist for final oocyte maturation and intensive luteal support. Hum Reprod.

[31] Humaidan P (2009). Luteal phase rescue in high-risk OHSS patients by GnRHa triggering in combination with low-dose HCG: a pilot study. Reprod BioMed Online.

[32] Humaidan P, Engmann L, Benadiva C (2015). Luteal phase supplementation after gonadotropin-releasing hormone agonist trigger in fresh embryo transfer: the American versus European approaches. Fertil Steril.

[33] Engmann L, Romak J, Nulsen J, Benadiva C, Peluso J (2011). In vitro viability and secretory capacity of human luteinized granulosa cells after gonadotropin-releasing hormone agonist trigger of oocyte maturation. Fertil Steril.

[34] Bodri D. Low-dose hCG supplementation after GnRH agonist triggering: Don’t be too quick on the trigger. Hum Reprod. 2013;9:2315–7. Oxford University Press. 10.1093/humrep/det125 23633554

[35] Meyer L, Murphy LA, Gumer A, Reichman DE, Rosenwaks Z, Cholst IN (2015). Risk factors for a suboptimal response to gonadotropin-releasing hormone agonist trigger during in vitro fertilization cycles. Fertil Steril.

[36] Bacchetti P (2010). Current sample size conventions: flaws, harms, and alternatives. BMC Med.

[37] Bacchetti P, Deeks SG, McCune JM (2011). Breaking free of sample size dogma to perform innovative translational research. Sci Transl Med.

